# A systems framework for implementing healthy food retail in grocery settings

**DOI:** 10.1186/s12889-023-17075-8

**Published:** 2024-01-09

**Authors:** Christina Zorbas, Miranda R. Blake, Andrew D. Brown, Anna Peeters, Steve Allender, Julie Brimblecombe, Adrian J. Cameron, Jill Whelan, Megan Ferguson, Laura Alston, Tara Boelsen-Robinson

**Affiliations:** 1 Global Centre for Preventive Health and Nutrition, Institute for Health Transformation, Deakin University, 1 Gheringhap Street, Geelong, VIC Australia; 2 Department of Nutrition and Dietetics, Monash University, 264 Ferntree Gully Rd, Notting Hill, VIC Australia; 3 School of Public Health, The University of Queensland, St Lucia, QLD Australia; 4 Deakin Rural Health, Faculty of Health, Deakin University, 1 Gheringhap Street, Geelong, VIC Australia

**Keywords:** Food retail, Supermarket, Food environments, Systems mapping

## Abstract

**Background:**

Food retailers can be reluctant to initiate healthy food retail activities in the face of a complex set of interrelated drivers that impact the retail environment. The Systems Thinking Approach for Retail Transformation (START) is a determinants framework created using qualitative systems modelling to guide healthy food retail interventions in community-based, health-promoting settings. We aimed to test the applicability of the START map to a suite of distinct healthy food marketing and promotion activities that formed an intervention in a grocery setting in regional Victoria, Australia.

**Methods:**

A secondary analysis was undertaken of 16 previously completed semi-structured interviews with independent grocery retailers and stakeholders. Interviews were deductively coded against the existing START framework, whilst allowing for new grocery-setting specific factors to be identified. New factors and relationships were used to build causal loop diagrams and extend the original START systems map using Vensim.

**Results:**

A version of the START map including aspects relevant to the grocery setting was developed (“START-G”). In both health-promoting and grocery settings, it was important for retailers to ‘Get Started’ with healthy food retail interventions that were supported by a proof-of-concept and ‘Focus on the customer’ response (with grocery-settings focused on monitoring sales data). New factors and relationships described perceived difficulties associated with disrupting a grocery-setting ‘Supply-side status quo’ that promotes less healthy food and beverage options. Yet, most grocery retailers discussed relationships that highlighted the potential for ‘Healthy food as innovation’ and ‘Supporting cultural change through corporate social responsibility and leadership’.

**Conclusions:**

Several differences were found when implementing healthy food retail in grocery compared to health promotion settings. The START-G map offers preliminary guidance for identifying and addressing commercial interests in grocery settings that currently promote less healthy foods and beverages, including by starting to address business outcomes and supplier relationships.

**Supplementary Information:**

The online version contains supplementary material available at 10.1186/s12889-023-17075-8.

## Background

Food systems in most countries have, over time, become increasingly focused on the manufacturing, marketing and sales of foods and beverages that are cheap, convenient and unhealthy [1]. The result has been widespread morbidity and mortality from obesity and other diet-related diseases [2]. Accordingly, global health experts have called for food systems transformations that prioritise human and planetary health in decision-making, and not just the profits of transnational corporations [3]. At the cornerstone of such food systems transformations are efforts to ensure the availability, accessibility, affordability and desirability of nutritious foods – all of which will require coherence in the actions of actors across the food system, including an increased responsibility by private sectors [3].

Food retailers are key actors in improving diets because they sit at the nexus of the food system and the people who purchase and consume food [4, 5]. Without changing retail food environments, significant improvements in diet-related population health will be difficult to achieve [6]. Food retailers can be reluctant to initiate healthy food retail (HFR) activities in the face of a complex set of interrelated drivers that impact the retail environment [7]. These include, but are not limited to, concerns about customer backlash, actions from multinational food producers focused on selling unhealthy food, and concerns about retail profitability [7, 8].

Despite these barriers, considerable advancement of HFR within specific contexts has been demonstrated – particularly in health-promoting settings such as health services [9–11], sports and recreation settings [12], and some local communities [13–15]. This work highlights the way in which public health practitioners in organisations with a health-promoting focus are well placed to engage and aid retailers in catalysing HFR changes. There are also a growing number of examples of local [16], state [17] and federal governments [18], and NGOs [19], developing, implementing, evaluating, and upscaling HFR initiatives in less health-promoting and commercially focused settings. Evidence from systematic reviews has found that supermarket interventions can be effective at improving the healthiness of customer purchases and diets [20–25]. Despite this, gaps in our knowledge remain regarding how HFR initiatives are best implemented by coalitions of stakeholders and sustained within these complex and highly competitive environments.

Few context-specific methodologies, theories, frameworks or models exist to guide implementation or evaluation of initiatives in the emerging research area of HFR. In particular, existing health promotion-based determinant frameworks may not be able to adequately guide planning and evaluation of initiatives in commercially focused complex systems because they do not comprehensively consider the tensions between health promotion and commercial objectives [7, 8]. Opportunities exist to address the limitations of current frameworks by drawing upon implementation and complexity science [26]. Implementation science provides guidance on planning implementation and evaluation of interventions through multiple generalisable frameworks. Systems thinking provides the techniques to develop empirical frameworks that understand complexity (for example, the complexity of the food system and its impact on consumers), and provide specific guidance on relationships of cause and effect through the lens of non-linearity, feedback and delays [27].

Based on the perspectives of retailers and other community stakeholders, the Systems Thinking Approach to Retail Transformation (START) map [28] sets out a systems-informed and retail-specific determinants framework for HFR implementation, which can be used to guide planning and evaluation. The START map describes 17 factors and five narratives that have been found to influence HFR implementation through four HFR evaluations within sports organisations, local government, and health services in Victoria, Australia [9–11, 29, 30]. Specifically, the START map describes change over time (dynamic complexity) to explain (i) how retailers gradually become less resistant to healthy food policies (as favourable business and health outcomes come to fruition); (ii) how easier HFR interventions are implemented before more complex options (associated with additional barriers to their implementation); (iii) how multiple means exist of obtaining organisational resourcing; (iv) the inter-relationships between retailer willingness to implement, and customer acceptability of, healthy retail initiatives; and (v) how retailer resistance increases when there is limited access to healthy options in the food supply [28].

Shifts towards HFR in grocery settings are needed to improve population health but rely on disrupting complex feedback loops that are yet to be fully understood. The aim of this study was to enhance our knowledge of the leverage points for implementing HFR initiatives in grocery settings. Our objective was to test the applicability of the START map to describe factors influencing the adoption, implementation and maintenance of several healthy food marketing and promotion initiatives that formed a HFR intervention in a grocery setting.

## Methods

### Study design and context

This study involved a qualitative approach with system dynamics as the main theoretical framework to understand the mechanisms that drive and hinder HFR in grocery settings [31]. This approach extends beyond traditional qualitative research (which seldom focuses on relationships) by describing the sum of the various parts in the grocery system as they relate to HFR. The research team has previously conducted HFR interventions and qualitative process evaluations in grocery store settings (MRB, AJC, JB, CZ, AP, AC) and have experience with systems mapping (ADB, JW, SA, LA).

### Data sources

A secondary dataset of stakeholder interviews evaluating HFR in grocery settings was deductively mapped against existing START map factors (sometimes defined as ‘variables’ and ‘determinants’ in the literature [32]) and factors identified by systematic reviews as relevant to implementing HFR in grocery settings. The research team conducted these interviews to evaluate the ‘Eat Well @ IGA’ intervention (described below). The interview data were also inductively coded for new factors.

#### The “Eat Well @ IGA” intervention and evaluation

The ‘Eat Well @ IGA’ randomised controlled trial (RCT) was a health-promoting intervention in 11 supermarkets in regional Victoria, Australia [33]. It consisted of several health-promoting changes to point-of-sale materials, including trolleys and basket signage, floor signs, brochures and social media, along with shelf-tags identifying healthier products according to the voluntary Australian government Health Star Rating nutrient profiling (front-of-pack labelling) system [34]. The distinct health promotion activities that were implemented were informed by a pilot study to support the transition. ‘Eat Well @ IGA’ was an initiative grounded in partnerships between several independent grocery retailers who fall under an umbrella organisation (Independent Grocers of Australia; IGA), local government, and researchers. An overview of the partnership development and intervention results [33], and a process evaluation of ‘Eat Well @ IGA’ have been previously published [34]. Following the intervention, eighteen semi-structured in-depth interviews were conducted by MRB with key stakeholders, including retail executives, store managers, marketing manager, store staff, research staff, and research liaison officer from the partnering local government. These ‘Eat Well @ IGA’ interviews aimed to understand how key stakeholders, predominantly retailers, perceived the process of implementing a HFR intervention [34]. Questions focused on understanding stakeholder involvement, benefits and limitations, and potential scalability and improvements with respect to the intervention.

The ‘Eat Well @ IGA’ interview data were comparable to the data that informed the development of the original START map in terms of scope and stakeholders interviewed (i.e., aims focused on evaluating the experiences and perspectives of retail managers and staff, and health-promotion staff involved in the implementation of HFR interventions, including barriers and facilitators; and dependence on co-design and partnerships). On this basis it was hypothesised that the content and scope was similar enough to allow comparison with ‘Eat Well @ IGA’ interviews. Compared to the ‘Eat Well @ IGA’ intervention (marketing and promoting healthier options), interventions underpinning the original START map focused on food availability and pricing interventions across four case studies. Differences in retail setting and customer base between the original START map and ‘Eat Well @ IGA’ datasets are explored in this analysis.

#### Sample selection

Interviews were sampled from the 18 original ‘Eat Well @ IGA’ interviews. Details of the complete interview dataset are published elsewhere [34]. Only in-depth retailer perspectives were included [28], resulting in 16 interviews that were considered relevant to the current research aim. Participants across six distinct roles (executive and marketing managers, produce and grocery managers, store owners, store managers, government representatives) were included. Two interviews with researchers were excluded to avoid biasing the findings towards factors and relationships that may have been of lesser concern to retailers. All participant interviews were analysed beyond data saturation to continue to test and collect examples of how the theoretical START map was adapted to grocery settings [35].

### Analytical approach

#### Analysis framework: the START map

This study was guided by the approach used to develop the original START map (described above and elsewhere [28], based on the work by Turner et al. [36]) and iterative consultation with all authors, including a systems methodology expert (ADB) [37]. ‘Eat Well @ IGA’ interviews were deductively coded against: (i) the predefined ‘relationships’ between factors in the START map (see glossary in Table 1; a ‘relationship’ between two factors describes a change in Factor 1 driving or resulting from a change in Factor 2) and (ii) eight factors identified from recent systematic reviews as potentially relevant to influencing HFR implementation in grocery settings (e.g., competition, opportunity costs, etc.) [38–40]. Newly identified relationships between factors were also inductively coded from the ‘Eat Well @ IGA’ interviews as they were identified.

**Table 1:** Glossary of systems terminology

**Key term**	**Representation in systems map**	**Explanation**
Factor	Factor 1	Standard factor, also referred to as a variable.
Shadow factor		A duplication of an existing factor in the map, used so that the map has fewer overlapping arrows and is easier to comprehend.
Positive causal relationship		A positive relationship between two factors, where an increase in Factor 1 results in an increase in Factor 2 and a decrease in Factor 1 results in a decrease in Factor 2.
Negative causal relationship		A negative relationship between two factors, where an increase in Factor 1 results in a decrease of Factor 2 and a decrease in Factor 1 results in an increase in Factor 2.
Stock		Builds and reduces slowly over time relative to the factors in the map. If stock inputs were to decrease, the stock itself would retain its magnitude, depending on other outputs and inputs.
Flow		Inputs to (arrow facing), or outputs from (arrow exiting), a stock, causing the stock to increase or decrease, respectively.
A “stock and flow”		“Engaging customer in healthy changes” is a flow into (i.e. builds) “Customer acceptability of healthy options”. We also have the flow of “Customer resistance to change”, which flows out (i.e. reduces) “Customer acceptability of healthy options”. If both “Engaging customers in healthy changes” and “Customer resistance to change” were to stop, the levels of “Customer acceptability of healthy options” would remain the same, as a stock.
Reinforcing loop		Pathway of relationships between factors that, when activated, amplifies the existing change in one direction.
Balancing loop		Pathway of relationships between factors that, when activated, has a goal seeking behaviour, directing the value of factors to specific values.

Reprinted from: Mapping factors associated with a successful shift towards healthier food retail in community-based organisations: A systems approach. Food Policy (101). Boelsen-Robinson T, Blake MR, Brown AD, Huse O, Palermo C, George NA, Peeters A. Copyright (2021), with permission from Elsevier

A subset of five interviews were used to pilot our coding approach (CZ). Two of these interviews were double coded by a second researcher who conducted the interviews (MRB). The analysis team (CZ, MRB, TBR) iteratively discussed similarities and differences in the relationships identified in the original START map and the grocery context. All coding was conducted using NVivo 12 qualitative data management software (see Additional file 1: Appendix 1 for the summary of methodological approach to developing the START-G map).

#### Adaption, validation, and synthesis of START-G map causal loops

The consistency of the relationships between factors affecting the implementation of HFR initiatives in grocery settings was tested beyond the subset of coded interviews by analysing the remaining eleven eligible ‘Eat Well @ IGA’ interviews. These relationships were then used to build causal loop diagrams and extend the original START map (converged using Vensim systems mapping software [41]). Under the guidance of a systems methods expert (ADB), the analysis team (CZ, TBR, MRB) iteratively adapted the START systems map; adding new factors, relationships, directions, and causal loops and cross-checking these with interview data, until consensus was achieved. The resulting map is called the Systems Thinking Approach for Retail Transformation in Grocery settings (START-G map).

The adapted START-G map and key narratives were presented to the local government representative that was involved in the original interviews for ‘Eat Well @ IGA’ (due to their oversight and frequent interactions with all stakeholders) to conduct internal validation. The interviewee was asked to identify any missing factors, relationships between factors, and whether any relationships between factors were inconsistent with their experiences. Interviewee feedback was considered and integrated by the analysis team. All authors provided feedback on the adapted START-G map and approved of the final version.

### Ethics

This study received ethical approval from the Deakin University Human Research Ethics Committee (HEAG-H 65_2015).

## Results

The key narratives underpinning the adapted START-G map are described below, including the relevance of original START map factors and new grocery-relevant narratives, ordered by the extent to which they were reported by retailers.

Most of the original START map factors and relationships were deductively identified in the ‘Eat Well @ IGA’ interview dataset. The adaptation process resulted in updated definitions for four factors. In addition, 13 new factors were inductively identified as being specifically relevant to the grocery context. These new factors were added to three thematic areas in the original START map (Table 2).

**Table 2:** New factors identified as relevant to the implementation of healthy food retail in grocery settings (based on the ‘Eat Well @ IGA’ intervention)

**Original START map theme**	**New START-G map factors**	**Definition**
*Customer, commercial viability and health-related outcomes (represented in green in the START-G map*^*a*^*)*	Total profit	Profits (i.e., revenue that exceeds costs) generated by the retailer from the sale of all products. Includes minimisation of loss from wastage of food not sold.
	Competitiveness of market position	The market share of a retailer relative to their competitors at a local and national level.
	Point-of-difference through healthy food environment	Ways in which retailers differentiate themselves from their market competitors to increase brand recognition and market share.
*Broader environmental influences (represented in dark blue in the START-G map)*	Prioritisation of public health nutrition by key stakeholders	Includes federal, state or local governments creating, monitoring and/or enforcing mandatory or voluntary policies, recommendations from international bodies such as WHO, and expectations to act on Environmental, Social, Governance (ESG) considerations by global investors.
	External support from workforce with healthy changes expertise	External practical support for, and expertise in, implementation of healthy food retail available to the organisation and/or retailer. Represents the workforce of health promotion practitioners with experience in healthy food retail implementation, can be from government departments, NGOs, universities, or other organisations.
	External recognition of healthy changes	Reward and recognition for healthy retail practices (e.g., accreditation, awards, media attention).
	Trust between retailer and public health stakeholders	Trust between the retailer and stakeholders working to improve public health, including health promotion practitioners and researchers, by convincing them to commit to healthy changes
	Relationship development between retail and public health representatives	Time invested in building the relationship. This should include a co-design process where all perspectives inform the design and implementation of healthy changes.
	Number and influence of food retail competitors making healthy changes	The number and success of other healthier food retail initiatives, including from retailers’ direct competitors.
	Number and influence of suppliers and manufacturers offering appropriate healthier alternatives	Number and size of suppliers and manufacturers creating healthier alternatives.
	Strength of contracts with suppliers favouring unhealthy foods	The extent to which retailers engage in contracts with suppliers and manufacturers to stock, sell and promote unhealthy foods and beverages. *Note: The food and beverage portfolios of these suppliers and manufacturers are typically unhealthy and thus the established contracts favour the ongoing availability and promotion of these products*.
*In-store food environment (represented in pink in the START-G map)*	Gap between proposed and current food retail environment	How substantial the perceived or actual difference is between the proposed healthier practices compared to current grocery practices. *Note: These practices currently favour the supply and promotion of unhealthy compared to healthy foods and beverages*.
	Healthiness of retail environment compared to current standard of practice	The extent to which current store practices promote healthier customer purchases, compared to its competitors. *Note: This can be a moving target depending on both the retailer’s own actions, and the actions of their competitors*.

^a^Colour coding of systems map in Figs. 2, 3, 4, 5 and 6: Retailer factors (orange), customer factors (green), organisational factors (light blue), in-store food environment factors (pink), broader environmental factors (dark blue) [28]

**Fig. 1:**
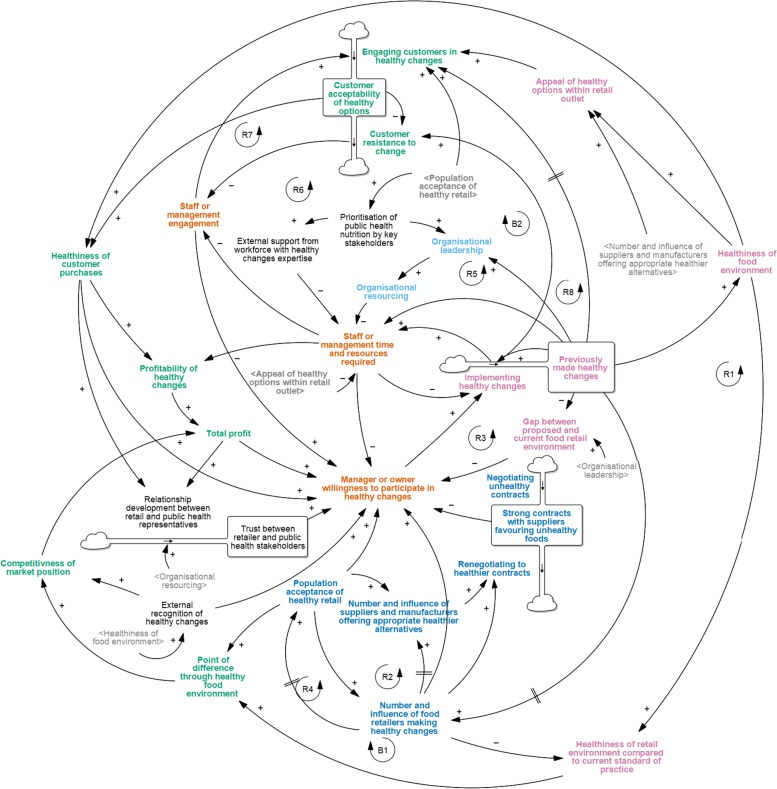
The full Systems Thinking Approach to Retail Transformation in Grocery settings (START-G map; see Table S1 for definitions of each factor). Reprinted from: Mapping factors associated with a successful shift towards healthier food retail in community-based organisations: A systems approach. Food Policy (101). Boelsen-Robinson T, Blake MR, Brown AD, Huse O, Palermo C, George NA, Peeters A. Copyright (2021), with permission from Elsevier

When adapting the original START map to include the new grocery-specific factors (START-G map; Fig. 1), two narratives underwent no or minor structural changes (*Get Started; Focus on the customer*) and one narrative underwent major structural changes to reflect how corporate leadership drives resource support (*Supporting cultural change through Corporate Social Responsibility (CSR) and leadership*). Two new narratives were incorporated into the START-G map (*Consider supply-side status quo; Healthy food as innovation*). Two original START narratives were not found to be applicable to the grocery context (*Work with suppliers to overcome time and resource barriers; Be prepared for diminishing return on investment*).

### Get Started

**Fig. 2:**
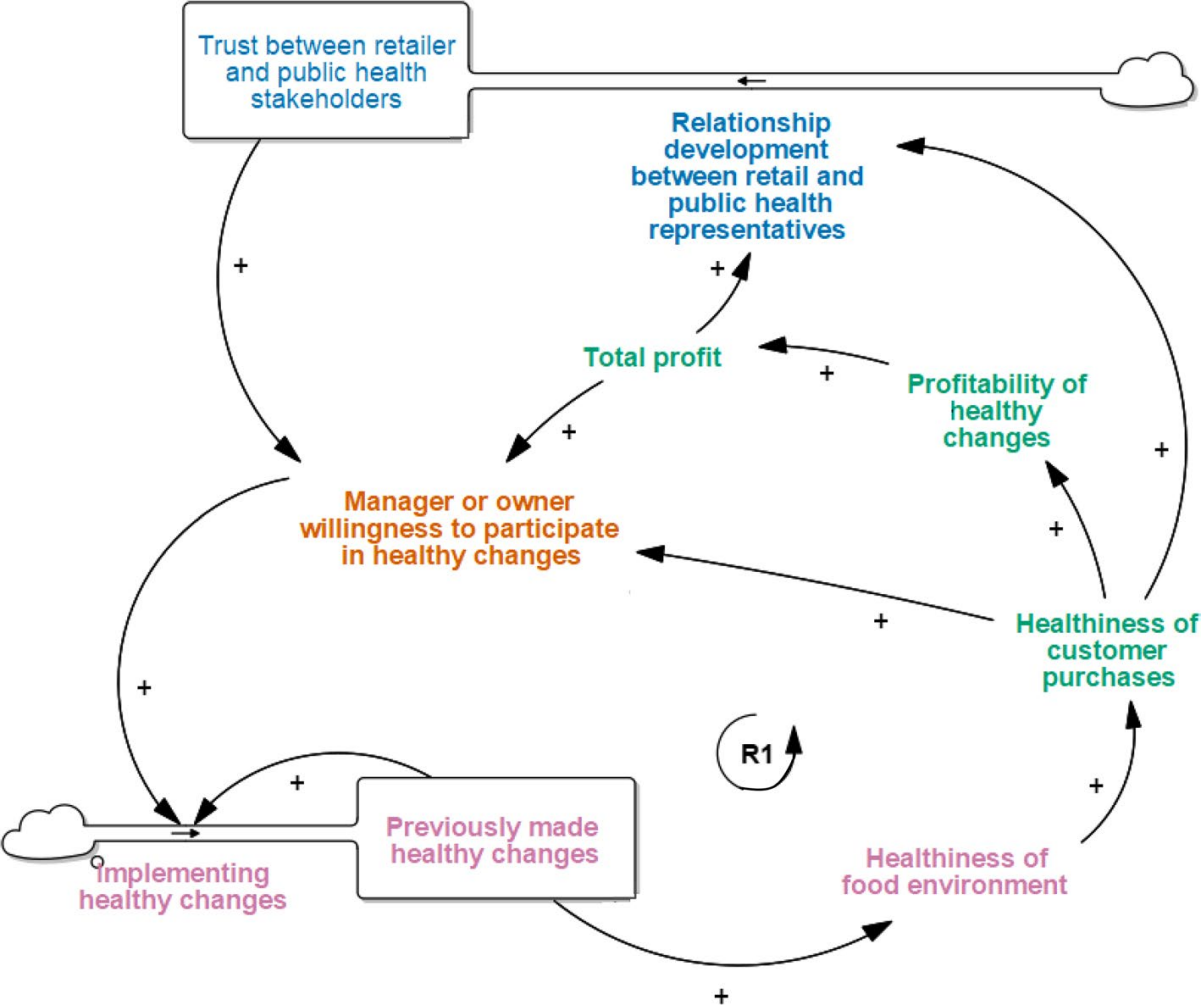
Get Started narrative in the START-G map. *This narrative is consistent with the original START map but has been updated to include grocery-specific considerations around total profit, relationship development between retail and public health representatives, and trust between retailer and public health stakeholders

Starting to implement healthy changes in food retail environments was one of the most frequently discussed START map narratives among IGA retailers (Fig. 2). In the context of ‘Eat Well @ IGA’, *previously made healthy changes* typically referred to the short-term trial intervention previously run by the grocery chain, researchers, and local government to inform the characteristics and feasibility of the RCT. Generating evidence on the impact of *previously made healthy changes* on *healthiness of customer purchases* and *profitability* was viewed as key to drive *manager or owner willingness to participate in healthy changes* in grocery settings. This is normal practice in grocery settings, with ‘proof of profitability’ widely recognised as necessary for the ongoing implementation of HFR:

*“…if you cut the [research] funding, basically, the first thing I would do is I would look at what I said before, the sales data… if the data shows that there’s an increase in sales, then we would actually have to look at how much is that increase in sales and then how much dollar value we’re actually getting” (Store owner, Supermarket A)*

Concerns about the *profitability of healthy changes* were discussed more holistically by grocery retailers compared to retailers in health promotion settings in the original START map; seen as a function of both the additional profits that may be generated from selling more healthy foods and beverages, and the loss in *total profit (new factor)* from the potential reduction in sales of unhealthy foods and beverages. Addressing retailer concerns during this process was also seen to *facilitate relationship development between retail and public health representatives (new factor)*. This process was perceived as essential to build *trust between retailer and public health stakeholders (new stock)* over time, thereby increasing retailer willingness to engage in HFR:

*“The constant contact with [the researchers and local government] was reassuring… we had regular meetings and we knew it was going to happen… it was pretty important that we actually did it and stuck to the timeframes and the guidelines, because everyone that was onboard, the stores and the staff, could really see the benefits.” (Store manager, Supermarket B)*

Although retailers were more willing to improve the *healthiness of the [grocery store] food environment* based on previously made changes, evidence, and the development of relationships, they also described how the typical use of the 4Ps of marketing (promotions, pricing, product, and placement) to drive the appeal of unhealthy products, hindered such efforts (see new narrative Consider supply-side status quo). For example:

*“unfortunately a lot of our promotions are geared around chocolate I suppose and chips and the more non-healthy side of the business. And our number one question is how is that going to affect our business and whether doing this program I suppose will affect our suppliers as well, because don’t forget we’ve never really been in that thought process. Yes we all talk about healthy living and we all talk about buying a healthier product, but in a business where most supermarkets are geared around your registers having chocolate on them and the impulse buy.” (Executive A)*

There was some indication that placing healthy, fresh produce in prominent store locations was also important for store profitability. The placement of fresh produce at the store entrance was seen to increase their appeal to customers, thereby increasing the healthiness of purchases and store profitability. One interviewee described how:

*“It’s the age-old rule of supermarkets where you know you strategically put stuff around the store to make sure people walk down aisles and stuff like that… we could do the same kind of thing and put stuff into the fresh departments to move sales” (Store manager, Store C)*

### Consider supply-side status quo

**Fig. 3:**
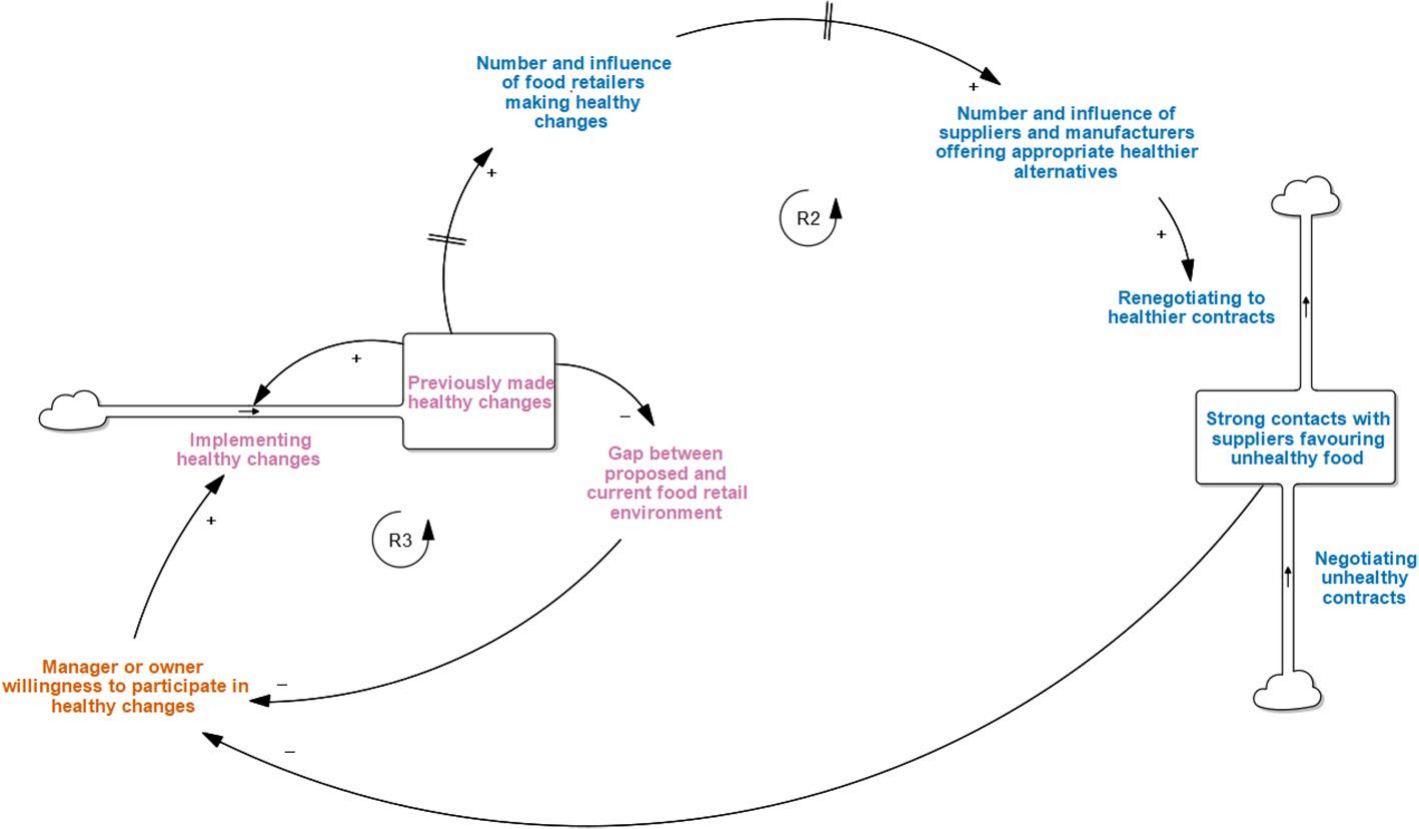
Consider Supply-side Status Quo narrative in the START-G map. *This adaptation to the original START map demonstrates grocery-specific considerations around supplier and retailer relationships

Within the IGA grocery settings, the *strength of contracts with suppliers favouring unhealthy foods (new factor)* was frequently discussed by retailers as a major factor that reduced *manager or owner willingness to participate in healthy changes* (Fig. 3).

*“It was a case of to decide on what the interventions would be and obviously there was a lot discussed, there were some that were presented that just wouldn’t work. It would just cause too many clashes associated with our suppliers” (Executive A)*

HFR changes that impacted product placement and promotion (often for less healthy foods and beverages) were thought to require engagement with suppliers to alter contracts and promotional agreements – which did not form part of the ‘Eat Well @ IGA’ intervention.

At the same time, the *previously made healthy changes* can have a delayed effect on increasing the *number and influence of food retailers making healthy changes (new factor)* which in turn, can potentially increase the *number and influence of suppliers and manufacturers offering (or promoting) appropriate healthier alternatives (new factor).* Previously made changes can also reduce the *gap between proposed (i.e., healthy) and current (i.e., less healthy) food retail environments (new factor)*. This gap describes the magnitude of the perceived or actual differences between the feasibility of the HFR intervention compared to current grocery practices and/or retailer reluctance to change.

*“Suppliers generally come in and they upsell their products... they will say, ‘Hey, next week we’ve got this on promotion. Can we do a display of it here?’… we would sort of say, ‘Look, I’m tight for space at the moment because I’m giving more space to healthier eating options’ and in their portfolio of products we might say, ‘Well hang on, you’ve actually got this, so how about we put that on instead of that and incorporate it into our Eat Well program?’ So we sort of sold it to them like that, which has worked out quite well.” (Store manager, Supermarket B)*

### Healthy food as innovation

**Fig. 4:**
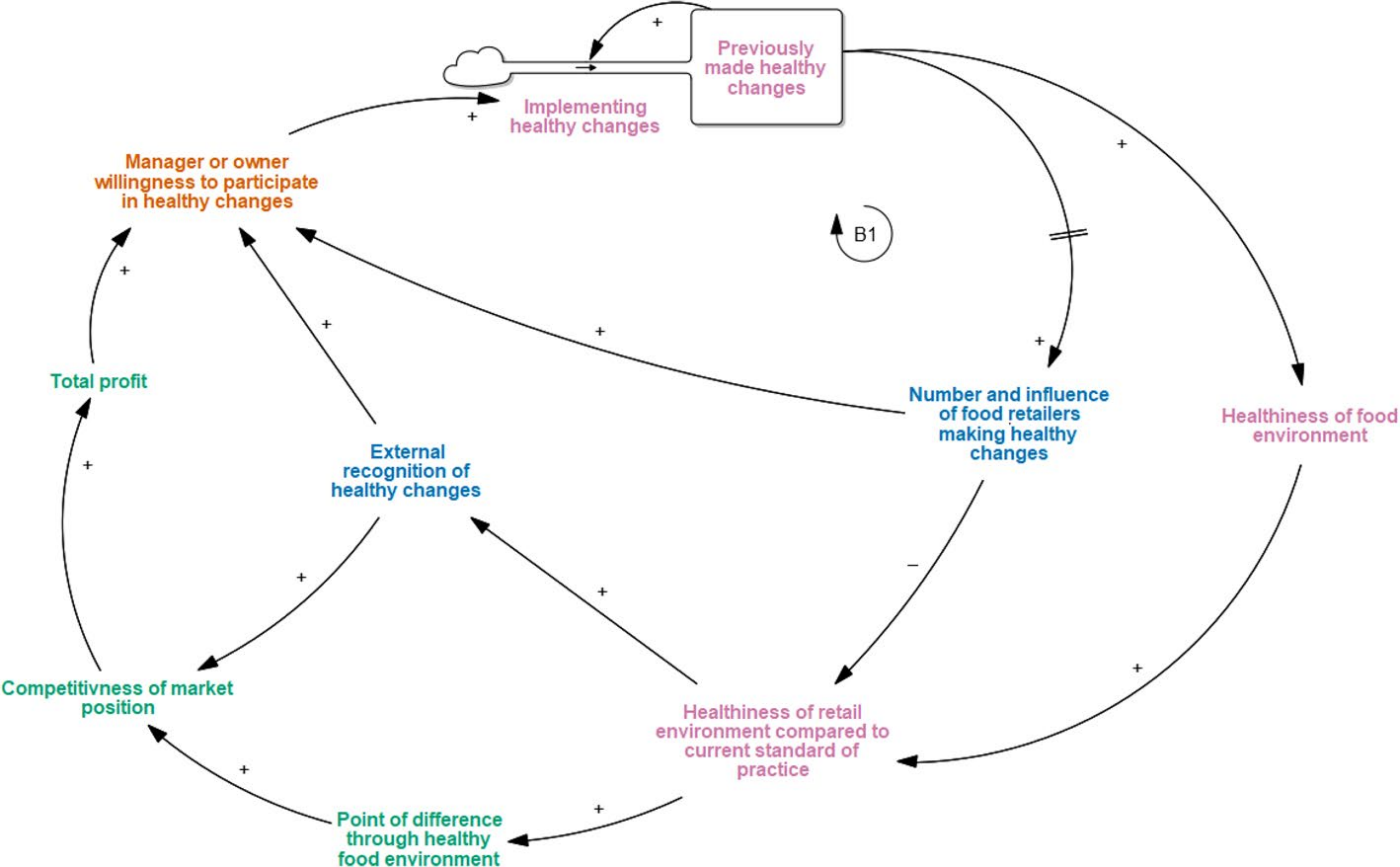
Healthy Food as Innovation narrative in the START-G map. *This adaptation to the original START map demonstrates grocery-specific considerations around using healthy food retail to create a point of different in the market

For most of the IGA stores involved, HFR was thought to be an innovative *point-of-difference (new factor)* from their competitors (Fig. 4). Interviewees reported that *implementing healthy changes* and having *previously made healthy changes* were opportunities for the IGA grocery stores to immediately increase the *healthiness of their food environments*, especially when compared to current grocery practices. The consequent point-of-difference was described as a way for IGA retailers to increase brand recognition by differentiating themselves as healthier retailers compared to their market competitors. HFR was also perceived to be one way that IGA could overcome negative perceptions about being more expensive and less aesthetically appealing than the larger supermarket chains:

*“For us it was a point-of-difference, I suppose, where - when you look at [the major supermarkets], they go out and they advertise - they claim to be the fresh food people, low prices or whatever, so for us it wasn’t really – we’re not claiming to be any of that. We’re just telling our customers we’ve got this program that they would benefit from if they came to our stores. Like, to encourage them to eat healthy and so forth. So it was a good point-of-difference. It wasn’t about price. It wasn’t about a claim of this or a claim of that. It’s more like you come to us and we can show you healthy eating options and so forth, so that was really good. That was a good point-of-difference.” (Store manager, Supermarket B)*

*External recognition of healthy changes (new factor)*, often through awards or media, was thought to be another benefit of, and therefore motivator for, HFR as innovation. A constant need to create a point of difference that was widely recognised was described by retailers as important for increasing the *competitiveness of their market position (new factor)* and thereby increasing *total profit (new factor)*. This consequently increased *manager or owner willingness to participate in healthy changes*. For one retailer:

*“I would say it’s important to try and encourage customers to eat not just for their benefit; also the benefit’s for us because obviously there’s more gross profit and all that type of stuff to be made in a lot of those products, particularly the fresh products.” (Store manager, Supermarket E)*

Yet, as HFR changes gain momentum, an increase in the *number and influence of food retailers making healthy changes (new factor)* ensues over time, which creates a new and healthier standard for grocery practices.

### Supporting cultural change through Corporate Social Responsibility (CSR) and leadership

**Fig. 5:**
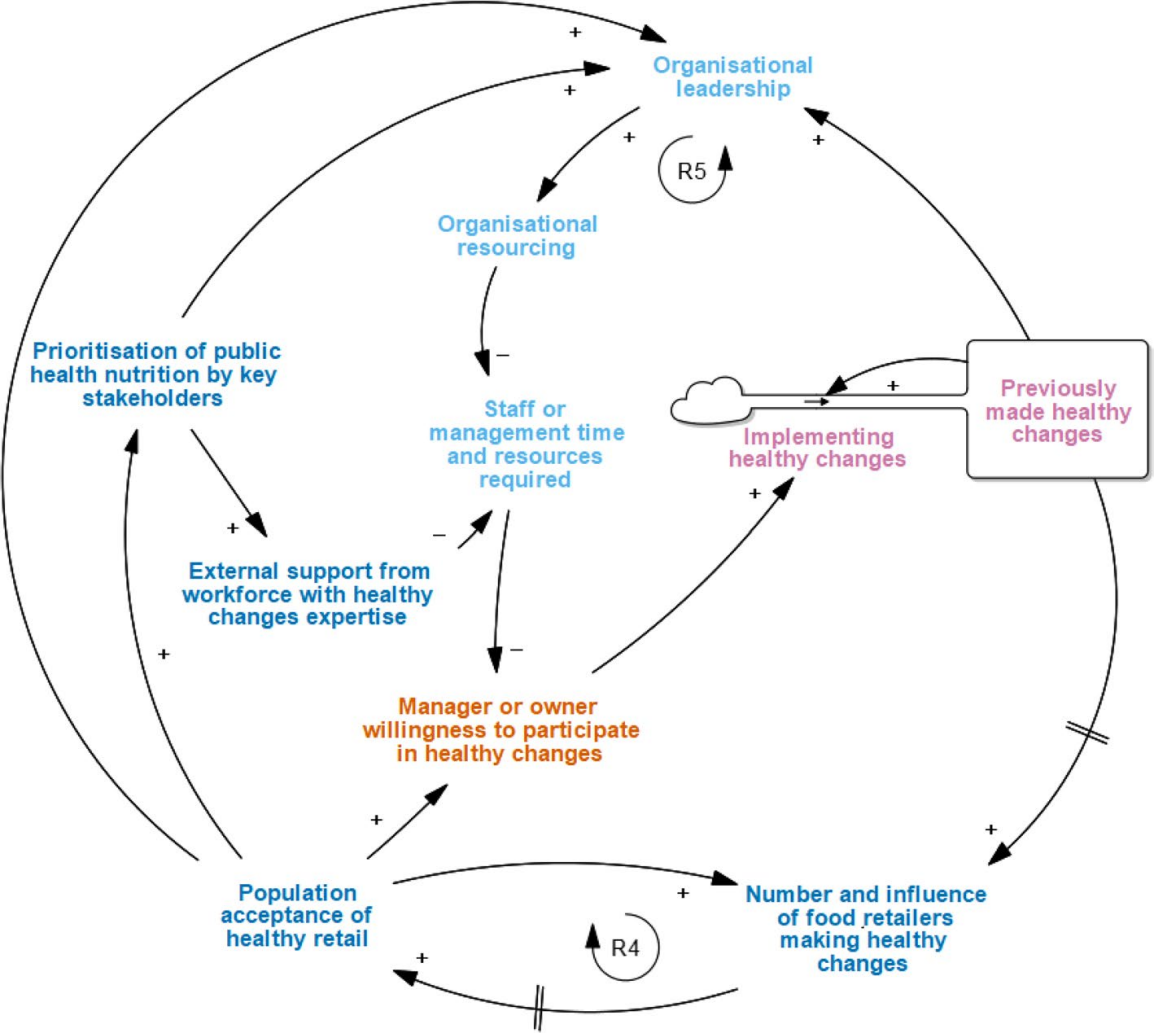
Supporting Cultural Change through Corporate Social Responsibility (CSR) and Leadership narrative in the START-G map. *This adaptation to the original START map demonstrates how healthy food retail can be motivated by CSR leadership

Retailers typically agreed that ‘Eat Well @ IGA’ provided an opportunity to be part of cultural change towards HFR (Fig. 5). START-G demonstrates how *previously made healthy changes* can reduce the *gap between proposed and current food retail environments* via health-promoting goal setting among retailers (i.e., organisational leadership), and an increase in *the number and influence of food retail competitors making healthy changes (new factor)*. These changes can increase *population acceptance of healthy retail* and create a reinforcing loop that supports cultural change in the long-term.

*“It would be good if they did bring it [‘Eat Well @ IGA’] out into all the stores. It would be because you could use that as an advert… we can’t really use it as an advertising point in our catalogues or anything because it’s only limited to a certain amount of stores. But then that opens everything up. Like more people can see that we are doing what we are doing and where everyone wants to be.” (Grocery manager, Supermarket F)*

In the context of this cultural change, grocery retailers exemplified *organisational leadership* through Corporate Social Responsibility (CSR; the inclusion of ethical, social and environmental concerns in business operations). CSR enabled the community to perceive that the retailers were not just operating for financial gain, but were also integral, trusted and loyal members of the community. The retailer (IGA) decision to align their values and practices with those that would promote community health and wellbeing, led to the prioritisation *organisational resources* towards HFR:

*“Well number one [value of the program], I suppose – probably the wrong term, being a responsible corporate citizen, I suppose.” (Executive B)*

In the ‘Eat Well @ IGA’ intervention, cultural change towards HFR also increased the *prioritisation of public health nutrition by key stakeholders (new factor)*. This in turn led to an increase in *external support from the workforce with healthy changes* expertise (new factor), including researchers and local government officers. The external support consequently increased *organisational resourcing* and reinforced store *manager or owner willingness to participate in the healthy changes*:

*“…to be honest, most of the stuff’s been driven by the guys from Deakin who come in each week, who follow up on missing flags and missing point of sale… we try our best to maintain any of the flags that fall off… that’s probably all that we’ve really done at store level…” (Store manager, Supermarket D)*

Whilst retailers typically recognised that some *time and additional resources* were required to maintain the shelf labels and other promotional materials, some also noted that the time costs could be easily absorbed into current staff duties and have minimal impacts on the *manager or owner willingness to participate*. This finding should however be considered within the context of the type of healthy retail intervention (e.g., marketing and promotion interventions).

### Focus on the customer

**Fig. 6:**
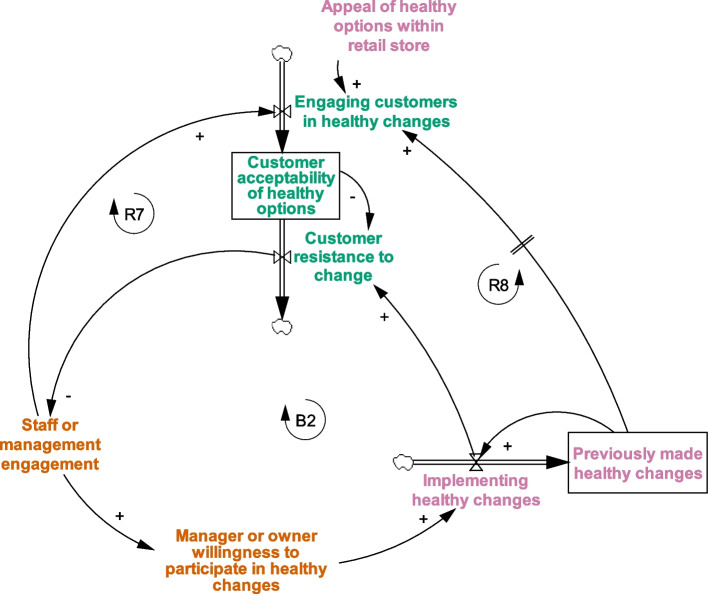
Focus on the Customer in the START-G map. *This narrative is consistent with the original START map but has been updated to include grocery-specific considerations around customer engagement with healthy food retail

Whilst grocery retailers generally thought that HFR should be driven by a focus on the customer (Fig. 6), this narrative was articulated to a lesser extent than in health promotion retail settings and was predominantly expressed by focusing on how the *healthiness of customer purchases* increases the *profitability of healthy changes*. *Customer resistance to change* was reported to be minimal during the intervention, with minimal impact on *outlet staff engagement*.

*“…some people quite liked it. The fact that I suppose it highlights some things and some things they probably wouldn’t have thought of… I wouldn’t say it was overwhelmingly positive]. But just as customers talk, they’d ask and that sort of stuff. Then generally speaking, it was fairly positive.” (Store manager, Supermarket F)*

To engage *customers in healthy changes* in the grocery setting, additional and regularly refreshed marketing and communications techniques were thought to be important to cut through the pervasive marketing and promotional techniques in current food environments. These additional engagement efforts included using print media, staff t-shirts, television, social media, and community spokespersons to promote the HFR changes to the public. One manager suggested how stores:

*“…just got to keep fresh, even if it’s just the images they’re using, something like that. I know cost-effective wise, you can’t change the trolley displays over every 3 months or 6 months. But there might be some new posters or fresh images” (Store manager, Supermarket G)*

## Discussion

### Summary of findings

This study used the Systems Thinking Approach for Retail Transformation (START) to expand our understanding of the potential leverage points that can support the implementation of HFR initiatives in grocery settings. Although the original START map was developed to reflect the implementation of a range of HFR interventions in health promoting settings, we found many aspects to be transferable to the ‘Eat Well @ IGA’ marketing and promotion intervention in independent grocers in Australia. In both health-promoting and grocery settings, it was important for retailers to (i) ‘Get Started’ with a HFR intervention and witness the achievement of favourable business and health outcomes, (ii) ‘Focus on the customer’ response (although grocery settings preferred to measure this through sales rather than customer feedback), and (iii) have sufficient leadership and resources to implement and maintain HFR initiatives [28]. Differences were identified in the grocery context through a greater focus on commercial risk, maintaining a competitive profit-focused market position, and the difficulties associated with disrupting a grocery supply chain that promotes less healthy food and beverage options – which are important considerations for health promotion practitioners in this context. Despite these challenges to implementing HFR, retailers also highlighted the potential for reframing HFR as a point-of-difference to increase market share and demonstrate leadership in the perceived cultural shift towards healthy eating.

The identification of commercial narratives aligns with several recently published systematic reviews that have captured the importance of understanding and measuring the impacts of HFR on business outcomes [38–40]. Our findings are further supported by a previous mixed-methods study involving 20 urban and non-urban, mostly independent, supermarket retailers in New York [42]. The authors similarly described how supplier contracts, customer demand, and market competitiveness influenced the types of foods that were available and marketed in grocery stores [42]. Additional efforts and resources were thought to be required to overcome these supply-side issues [42]. Despite these commercial challenges typically favouring less healthy food and beverage options, the grocery retailers interviewed were found to have the capacity and autonomy to lead HFR changes [42]. Similarly, we found that Australian grocery retailers were willing to contribute to promoting the health of their communities and capitalise on the cultural shift towards healthy eating.

Even though retailers may have good intentions to promote community health, the power interplay within the food system that creates barriers for retailers to shift towards HFR should be of primary concern. There is substantial recognition of how conglomerate-power, driven by commercial, political, economic, and societal factors, fosters environments that do not prioritise health across food systems [43]. The rapidly evolving research on the commercial determinants of population diets further demonstrates how marketing, supply chains, and corporate citizenship are all core channels that industries prioritise to drive corporate growth [44]. Indeed, we found that marketing unhealthy foods and beverages was a core business strategy in the grocery setting, alongside efforts to appear to be a business that cares about and is integral to the community (i.e., corporate citizenship). Previous studies have also emphasised that supermarkets often hold significant power in their contracts with suppliers, that can often disadvantage both the supplier and consumers, and favour the promotion of unhealthy foods and beverages [45]. Conversely, in our study, retailers typically indicated that they were bound by supplier contracts, particularly by companies who provide and want to promote unhealthy products. These contracts can create an external governance structure that constrains what aspects of food retail can be changed by retailers like IGA.

Although the focus of our study was on the retailer, our use of qualitative systems dynamics emphasised that there is also a need for intervention at the external supplier level to further facilitate real change in retail food environments. Our systems lens enabled us to broadly capture the grocery context and examine more nuanced interactions between key factors affecting HFR than traditional qualitative research methods. Systems Thinking has been used to effectively inform the implementation of community-level obesity prevention interventions in Australia [46]. The causal loop diagrams that we have created can be practically applied in the real-world to guide the complex implementation of HFR, ensuring the perspectives of retailers are considered. As with the original START map, we once again demonstrate how HFR implementation dynamically changes over time and how these changes can be addressed [31].

### Implications for future research and practice

Each of our key learnings, captured through the START-G implementation narratives, can be used to guide implementation of HFR in grocery contexts similar to ‘Eat Well @ IGA’. Firstly, with strong supplier contracts and perceived commercial barriers supporting the status quo of unhealthy food environments, stakeholders who want to implement HFR (e.g. public health practitioners) should focus on co-creating interventions with retailers to understand what is considered feasible within their grocery context [47]. Secondly, retailers may perceive HFR as an opportunity to differentiate their store offerings in the grocery market, suggesting that framing HFR as a business opportunity is likely to be more effective at engaging retailers than traditional health perspectives [38, 39]. Third, a retailer may be more likely to perceive HFR as a business opportunity if they can clearly differentiate their brand in the market and there is evidence there will be no adverse impacts to store profitability, highlighting the need to build the business case to support HFR in grocery settings [38].

Our fourth narrative demonstrates how the relationships between grocery suppliers and retailers should be viewed as interdependent. That is, grocery retailers such as IGA have the power to influence the production and distribution of healthy options, and suppliers can also influence healthy food environments through the product availability, promotional/ placement contracts, and reformulation [45]. Policy regulation of food environments and the supply-side of the food system is therefore essential to support the feasibility and effectiveness of HFR, as recommended by the World Health Organization [48]. Finally, grocery settings influence our food cultures, social norms around food purchasing and consumption, and thus the acceptability of HFR [45]. Grocery stores can use the increasing public acceptability of HFR and market competitiveness to accelerate and be at the forefront of our cultural movement towards the normalisation of HFR, including through the inclusion of more robust nutrition targets in their own policies [49].

Additional research is required to continue to refine and test the START-G map. This should include understanding if these lessons and frames can be used to upscale the implementation of HFR to larger grocery contexts in Australia and around the world and START-G’s applicability to a variety of health promotion approaches. Moreover, quantifying the effects of the different systems pathways, including the associated costs, would also help navigate HFR transformations in the real-world. Considerable scope exists to investigate the methods underpinning quantitative HFR systems simulation models into the future. Finally, our research team has demonstrated that tools and step-by-step guides are needed to translate systems frameworks into practice [50]. The development of such tools to impactfully implement HFR in grocery settings will necessitate comprehensive co-design processes that are conducted in collaboration with retailers, researchers and health promotion practitioners. Within these tools, it is important to identify successful case studies and cocreate strategies for managing trade-offs.

### Strengths and limitations

The START and START-G maps were derived from secondary data sources using post-hoc analyses of robust process evaluations of HFR in health promotion and grocery settings. Ideally, a priori interview questions with a range of stakeholders involved in the implementation of HFR initiatives should explicitly test the factors and constructs of the START maps. Further, using stakeholder interview analysis to build causal loop diagrams has been found to result in stronger alignment between the factors and relationships identified by participants in applied settings, compared to conducting group model building exercises [51]. However, there are limited existing interventions being conducted in grocery settings in general, both in Australia and more broadly. Therefore, we sought to test the applicability of the START map to grocery settings using the best available datasets, drawing upon studies with comparable research aims, stakeholders, and discussion guides.

It should also be noted that the type of HFR interventions varied between studies used to inform the START map (availability-based changes and a co-designed intervention increasing the price of unhealthier beverage options) and START-G map (a co-designed intervention to improve the marketing and promotion of healthy food and beverage options), and are likely to vary in the real-world and literature [20]. Nonetheless, the START and START-G maps do not intend to differentiate between different types of HFR interventions (availability versus price) or study designs (RCT versus natural experiment).

## Conclusions

If food retail settings, in particular grocery food retail settings, can be transformed towards HFR, they hold extensive potential for improving population health. Whilst HFR in grocery settings will need to work through similar processes as those that have been previously mapped in health promoting settings within public institutions, the START-G map suggests that some additional factors and relationships should be considered. A stronger emphasis on reframing and managing commercial interests should be of primary concern when implementing HFR in grocery settings. Additional studies should use this knowledge to guide the implementation of scalable grocery interventions that promote both customer health and retailer interests into the future.

### Supplementary Information

**Additional file 1: Appendix 1.** Summary of methodological approach to developing the START-G map. **Figure S1.** Summary of coding and validation process to create a systems map to describe the causal determinants of the adoption, implementation and maintenance of a healthy food marketing and promotion intervention in a grocery setting. **Table S1.** Description of factors included in final abstracted START-G framework of factors affecting successful implementation of healthy food retail initiatives in grocery settings.

## Data Availability

The datasets generated and/or analysed during the current study are not publicly available as participants did not provide their consent for this data to be shared beyond the research team but de-identified data are available from the corresponding author on reasonable request.

## Abbreviations

CSR: Corporate Social Responsibility
HFR: Healthy Food Retail
IGA: Independent Grocers Australia
RCT: Randomised Controlled Trial
START: Systems Thinking Approach for Retail Transformation
START-G: Grocery extension of Systems Thinking Approach for Retail Transformation

## References

[1] Swinburn BA, Sacks G, Hall KD, McPherson K, Finegood DT, Moodie ML (2011). The global obesity pandemic: shaped by global drivers and local environments. Lancet.

[2] Murray CJL, Aravkin AY, Zheng P, Abbafati C, Abbas KM, Abbasi-Kangevari M (2020). Global burden of 87 risk factors in 204 countries and territories, 1990–2019: a systematic analysis for the Global Burden of Disease Study 2019. Lancet.

[3] Webb P, Benton TG, Beddington J, Flynn D, Kelly NM, Thomas SM (2020). The urgency of food system transformation is now irrefutable. Nat Food.

[4] Caspi CE, Lenk K, Pelletier JE, Barnes TL, Harnack L, Erickson DJ (2017). Association between store food environment and customer purchases in small grocery stores, gas-marts, pharmacies and dollar stores. Int J Behav Nutr Phys Act.

[5] Glanz K, Bader MD, Iyer S (2012). Retail grocery store marketing strategies and obesity: an integrative review. Am J Prev Med.

[6] Afshin A, Sur PJ, Fay KA, Cornaby L, Ferrara G, Salama JS (2019). Health effects of dietary risks in 195 countries, 1990–2017: a systematic analysis for the Global Burden of Disease Study 2017. Lancet.

[7] Gravlee CC, Boston PQ, Mitchell MM, Schultz AF, Betterley C (2014). Food store owners’ and managers’ perspectives on the food environment: an exploratory mixed-methods study. BMC Public Health.

[8] Gittelsohn J, Franceschini MC, Rasooly IR, Ries AV, Ho LS, Pavlovich W (2008). Understanding the food environment in a low-income urban setting: implications for food store interventions. J Hunger Environ Nutr.

[9] Boelsen-Robinson T, Backholer K, Corben K, Blake MR, Palermo C, Peeters A (2017). The effect of a change to healthy vending in a major Australian health service on sales of healthy and unhealthy food and beverages. Appetite.

[10] Blake MR, Peeters A, Lancsar E, Boelsen-Robinson T, Corben K, Stevenson CE (2018). Retailer-led sugar-sweetened beverage price increase reduces purchases in a hospital convenience store in Melbourne, Australia: a mixed methods evaluation. J Acad Nutr Diet.

[11] Boelsen-Robinson T, Blake MR, Backholer K, Hettiarachchi J, Palermo C, Peeters A (2019). Implementing healthy food policies in health services: a qualitative study. Nutr Diet.

[12] Boelsen-Robinson T, Orellana L, Backholer K, Kurzeme A, Jerebine A, Gilham B, et al. Change in drink purchases in 16 Australian recreation centres following a sugar-sweetened beverage reduction initiative: an observational study. BMJ Open. 2020;10(3):e029492. Accepted 3/01/2020. 10.1136/bmjopen-2019-029492 PMC7059533 32139479

[13] Alston L, Crooks N, Strugnell C, Orellana L, Allender S, Rennie C (2019). Associations between school food environments, body mass index and dietary intakes among regional school students in Victoria, Australia: a cross-sectional study. Int J Environ Res Public Health.

[14] Whelan J, Love P, Millar L, Allender S, Bell C (2018). Sustaining obesity prevention in communities: a systematic narrative synthesis review. Obes Rev.

[15] Fehring E, Ferguson M, Brown C, Murtha K, Laws C, Cuthbert K (2019). Supporting healthy drink choices in remote Aboriginal and Torres Strait Islander communities: a community-led supportive environment approach. Aust N Z J Public Health.

[16] Boelsen-Robinson T, Peeters A, Thow A, Hawkes C. Barriers and facilitators to delivering a healthier food outlet initiative: perspectives from local authorities. Public Health Nutr. 2020. 10.1017/S1368980020002323 PMC10195441 32895071

[17] Healthy food and drink in NSW health facilities for staff and visitors framework. New South Wales Ministry of Health. North Sydney: NSW Ministry of Health; 2017.

[18] Tay Z, Whitton C, van Dam R, Rebello S. Food-EPI Singapore Report 2018. Benchmarking policies in creating healthier food environments: current policies and recommended actions. Singapore: National University of Singapore; 2018.

[19] Heart Foundation. Healthier oils program. 2018. Available from: https://www.heartfoundation.org.au/programs/healthier-oils-program.

[20] Cameron A, Charlton E, Ngan W, Sacks G (2016). A systematic review of the effectiveness of supermarket-based interventions involving product, promotion, or place on the healthiness of consumer purchases. Curr Nutr Rep.

[21] Golding SE, Bondaronek P, Bunten AK, Porter L, Maynard V, Rennie D (2022). Interventions to change purchasing behaviour in supermarkets: a systematic review and intervention content analysis. Health Psychol Rev.

[22] Harbers MC, Beulens JWJ, Rutters F, de Boer F, Gillebaart M, Sluijs I (2020). The effects of nudges on purchases, food choice, and energy intake or content of purchases in real-life food purchasing environments: a systematic review and evidence synthesis. Nutr J.

[23] Hartmann-Boyce J, Bianchi F, Piernas C, Payne Riches S, Frie K, Nourse R (2018). Grocery store interventions to change food purchasing behaviors: a systematic review of randomized controlled trials. Am J Clin Nutr.

[24] Mah CL, Luongo G, Hasdell R, Taylor NGA, Lo BK (2019). A systematic review of the effect of retail food environment interventions on diet and health with a focus on the enabling role of public policies. Curr Nutr Rep.

[25] Shaw SC, Ntani G, Baird J, Vogel CA (2020). A systematic review of the influences of food store product placement on dietary-related outcomes. Nutr Rev.

[26] Braithwaite J, Churruca K, Long JC, Ellis LA, Herkes J (2018). When complexity science meets implementation science: a theoretical and empirical analysis of systems change. BMC Med.

[27] Sterman JD (2006). Learning from evidence in a complex world. Am J Public Health.

[28] Boelsen-Robinson T, Blake MR, Brown AD, Huse O, Palermo C, George NA (2021). Mapping factors associated with a successful shift towards healthier food retail in community-based organisations: a systems approach. Food Policy.

[29] Peeters A, Huse O (2017). City of Melbourne: sports and recreation centre healthy food retail case study.

[30] Blake MR, Ryan A, Peeters A (2018). Healthy eating in the west- follow-up evaluation: city of Melton.

[31] Sterman JD (2000). Business dynamics: systems thinking and modeling for a complex world.

[32] Allender S, Owen B, Kuhlberg J, Lowe J, Nagorcka-Smith P, Whelan J (2015). A community based systems diagram of obesity causes. PLoS One.

[33] Blake MR, Sacks G, Marshall J, Brown AK, Cameron AJ, Potvin L, Jourdan D (2022). A successful intervention research collaboration between a supermarket chain, the local government, a non-governmental organization and academic researchers: the Eat Well @ IGA Healthy supermarket partnership. Global handbook of health promotion research, vol 1: mapping health promotion research.

[34] Blake MR, Sacks G, Zorbas C, Marshall J, Orellana L, Brown AK (2021). The ‘Eat Well @ IGA’ healthy supermarket randomised controlled trial: process evaluation. Int J Behav Nutr Phys Act.

[35] Braun V, Clarke V. To saturate or not to saturate? Questioning data saturation as a useful concept for thematic analysis and sample-size rationales. Qual Res Sport Exer Health. 2021;13:(2):201–16.

[36] Turner BL, Kim H, Andersen DF (2013). Improving coding procedures for purposive text data: researchable questions for qualitative system dynamics modeling. Syst Dyn Rev.

[37] Owen B, Brown AD, Kuhlberg J, Millar L, Nichols M, Economos C (2018). Understanding a successful obesity prevention initiative in children under 5 from a systems perspective. PLoS One.

[38] Blake MR, Backholer K, Lancsar E, Boelsen-Robinson T, Mah C, Brimblecombe J (2019). Investigating business outcomes of healthy food retail strategies: a systematic scoping review. Obes Rev.

[39] Middel CNH, Schuitmaker-Warnaar TJ, Mackenbach JD, Broerse JEW (2019). Systematic review: a systems innovation perspective on barriers and facilitators for the implementation of healthy food-store interventions. Int J Behav Nutr Phys Act.

[40] Houghtaling B, Serrano EL, Kraak VI, Harden SM, Davis GC, Misyak SA (2019). A systematic review of factors that influence food store owner and manager decision making and ability or willingness to use choice architecture and marketing mix strategies to encourage healthy consumer purchases in the United States, 2005–2017. Int J Behav Nutr Phys Act.

[41] Ventana Systems. Vensim PLE. 7.3 ed. Harvard: Ventana Systems, Inc; 1988–2017.

[42] Martinez O, Rodriguez N, Mercurio A, Bragg M, Elbel B (2018). Supermarket retailers’ perspectives on healthy food retail strategies: in-depth interviews. BMC Public Health.

[43] Leach M, Nisbett N, Cabral L, Harris J, Hossain N, Thompson J (2020). Food politics and development. World Dev.

[44] Kickbusch I, Allen L, Franz C (2016). The commercial determinants of health. Lancet Glob Health.

[45] Pulker CE, Trapp GSA, Scott JA, Pollard CM. What are the position and power of supermarkets in the Australian food system, and the implications for public health? A systematic scoping review. Obes Rev. 2017. 10.1111/obr.12635 29193744

[46] Allender S, Brown AD, Bolton KA, Fraser P, Lowe J, Hovmand P (2019). Translating systems thinking into practice for community action on childhood obesity. Obes Rev.

[47] Mackenbach J, Middel C, Beulens J, Lakerveld J, Broerse J, Schuitmaker-Warnaar TJ (2020). Towards an impactful intervention in a food retail setting - insights from transition management. Eur J Public Health.

[48] WHO. Tackling NCDs: best buys. Geneva: World Health Organization; 2017.

[49] Pulker CE, Trapp GSA, Scott JA, Pollard CM (2019). The nature and quality of Australian supermarkets’ policies that can impact public health nutrition, and evidence of their practical application: a cross-sectional study. Nutrients.

[50] Rozman M, Annois B, Johnson K, Livaditis C, Blake MR, Boelsen-Robinson T, Hedley J, Rosewarne E, Hobbs V (2023). Healthy retail toolkit, February 2023.

[51] Valcourt N, Walters J, Javernick-Will A, Linden K (2020). Assessing the efficacy of group model building workshops in an applied setting through purposive text analysis. J Syst Dyn Rev.

