# Supplementary material for: A systems framework for implementing healthy food retail in grocery settings
Source: BMC Public Health. 2024 Jan 9;24:137. doi: 10.1186/s12889-023-17075-8 (PMC10777568; doi:10.1186/s12889-023-17075-8)
Supplement: Supplementary file 1 — Additional file 1: Appendix 1. Summary of methodological approach to developing the START-G map. Figure S1. Summary of coding and validation process to create a systems map to describe the causal determinants of the adoption, implementation and maintenance of a healthy food marketing and promotion intervention in a grocery setting. Table S1. Description of factors included in final abstracted START-G framework of factors affecting successful implementation of healthy food retail initiatives in grocery settings. [file 12889_2023_17075_MOESM1_ESM.docx]

#### **A systems framework for implementing healthy food retail in grocery settings – Supplement**

**Appendix 1- Summary of methodological approach to developing the START-G map**

**Sampling and preliminary coding**

Analysis team identified 5 ‘core’ retailer interviews from n=18 interviews focused on evaluating the ‘Eat Well @ IGA intervention’.

Core interviews deductively coded against original START map and inductively coded for new factors and relationships (double coding 2 interviews).

Data dictionary updated.

**Identifying and validating core relationships and narratives**

Coding used by analysis team to identify core relationships and narratives between key factors.

Remaining eligible interviews (n=10 retailers included, n=3 researchers excluded) coded to validate findings.

**Modifying causal loop diagrams**

New relationships between factors added to existing START map by analysis team.

Analysis team iteratively modified causal loop diagrams (by adding/removing new factors and cross-checking changes against interview data) until consensus was attained that the map and narratives reflected core discussions.

New factors grouped to original START map themes (i.e. domain of retail influence – see Table 2).

New narratives and modifications to original START narratives detailed in text.

**Final validation**

All authors cross-checked core causal loops and narratives.

Internal validity testing conducted with research officer participating in original interviews.

**Figure S1. Summary of coding and validation process to create a systems map to describe the causal determinants of the adoption, implementation and maintenance of a healthy food marketing and promotion intervention in a grocery setting.**

**Table S1. Description of factors included in final abstracted START-G framework of factors affecting successful implementation of healthy food retail initiatives in grocery settings**

| **Factor** | **Description** |
| --- | --- |
| **Original START map framework** | |
| Manager or owner willingness to participate in healthy changes | Store owner or management's support for the initiative, including perceived risk of the intervention. Store owner or manager broadly refers to the person who makes strategic decisions at the retail site level, and may differ depending on the size of the store and whether they are owned as a chain of outlets. |
| Outlet staff and manager and owner time and resources required | Time, cost and resourcing for retailer to implement and maintain healthy changes. Includes potential additional time and cost associated with buying and preparing healthier options. |
| Outlet staff engagement | Retail site staff training, knowledge, capacity and enthusiasm for healthy initiative. Includes those who may be tasked with implementation of healthy initiative and may have direct contact with customers but do not have high-level, decision-making power. May include a 'champion' who drives the changes. |
| Profitability of healthy changes | Anticipated and actual commercial viability outcomes of the healthy change, including factors such as wastage of perishable food and cost of items. |
| Healthiness of customer purchases | Public health status measures such as healthiness of purchases. |
| Customer acceptability of healthy options | Customer awareness and responses to initiative, as well as retailer concern with and reaction to customer responses. Popularity of healthy alternatives, customer preference for healthy alternatives, customer nutrition knowledge. |
| Customer resistance to change | Customer dissatisfaction with, and resistance to, changes in the in-store food retail environment. |
| Engaging customers in healthy changes* | Deliberate actions to communicate and promote support for and interest in the healthy changes among customers. May be driven internally by retailer or externally, such as by a health promotion practitioner. |
| Organisational leadership* | Organisational desire for change, high-level/executive support/mandate. Desire to be seen as a leader in the space of health. Prioritisation of health goals within organisation including allocation of resources towards change. |
| Organisational resourcing | Practical support given by the organisation, such as a dedicated health promotion staff member and their actions, and the building of relationships between retailer and organisational staff. Internal organisational funding dedicated to support implementation. Includes the skills and experience of the health promotion staff e.g. their understanding of primary prevention, partnership and relationship skills, willingness to step out of their role, and food service and dietetic skills. |
| Previously made healthy changes (a stock**^Ɨ^**) | Extent and maintenance of previously implemented changes to the healthiness of the in-store food environment (e.g. price of water previously lowered). |
| Implementing healthy changes | The action of making healthy changes to the in-store food environment (e.g. lowering the price of water). |
| Healthiness of the food environment* | Healthiness of the in-store food environment of the retailer in question, which may include the availability, pricing, promotion and placement of healthy compared to less healthy alternatives. |
| Appeal of healthy options within retail outlet | Business practices designed to enhance the experience of the target customer. Includes factors affecting the appeal of the store and healthy alternatives within the store, such as store aesthetics, convenience, availability of specialist diet or culturally appropriate alternatives etc. |
| Population acceptance of healthy retail* | Broader acceptability of similar healthier food retail initiatives in the community. Consumer expectations for healthier food provision both for the specific organisation and more broadly, where expectations for healthy food provision may be enhanced when in relation to a health-promoting or community organisation. |
| Supply of appropriate healthy alternatives | Number of, ease of access to and acceptable supply options to retailer. Healthy alternatives that are cost effective, palatable, and acceptable alternatives for customers to unhealthier options. Encapsulates the 5 dimensions of food access: accessibility, affordability, availability, accommodation and acceptability. |
| **New START-G framework (additional factors)** | |
| Total profit | Profits (i.e., revenue that exceeds costs) generated by the retailer from the sale of all products. Includes minimisation of loss from wastage of food not sold. |
| Competitiveness of market position | The market share of a retailer relative to their competitors at a local and national level. |
| Point-of-difference through healthy food environment | Ways in which retailers differentiate themselves from their market competitors to increase brand recognition and market share. |
| Prioritisation of public health nutrition by key stakeholders | Includes federal, state or local governments creating, monitoring and/or enforcing mandatory or voluntary policies, recommendations from international bodies such as WHO, and expectations to act on Environmental, Social, Governance (ESG) considerations by global investors. |
| External support from workforce with healthy changes expertise | External practical support for, and expertise in, implementation of healthy food retail available to the organisation and/or retailer. Represents the workforce of health promotion practitioners with experience in healthy food retail implementation, can be from government departments, NGOs, universities, or other organisations. |
| External recognition of healthy changes | Reward and recognition for healthy retail practices (e.g. accreditation, awards, media attention). |
| Trust between retailer and public health stakeholders | Trust between the retailer and stakeholders working to improve public health, including health promotion practitioners and researchers, by convincing them to commit to healthy changes |
| Relationship development between retail and public health representatives | Time invested into building the relationship. This should include a co-design process where all perspectives inform the design and implementation of healthy changes. |
| Number and influence of food retail competitors making healthy changes | The number and success of other healthier food retail initiatives, including from retailers’ direct competitors. |
| Number and influence of suppliers and manufacturers offering appropriate healthier alternatives | Number and size of suppliers and manufactures creating and promoting healthier alternatives. |
| Strength of contracts with suppliers favouring unhealthy foods | The extent to which retailers engage in contracts with suppliers and manufacturers to stock, sell and promote unhealthy foods and beverages. *Note: The food and beverage portfolios of these suppliers and manufacturers are typically unhealthy and thus the established contracts favour the ongoing availability and promotion of these products.* |
| Gap between proposed and current food retail environment | How substantial the perceived or actual difference is between the proposed healthier practices compared to current grocery practices. *Note: These practices currently favour the supply and promotion of unhealthy compared to healthy foods and beverages.* |
| Healthiness of retail environment compared to current standard of practice | The extent to which current store practices promote healthier customer purchases, compared to its competitors. *Note: This can be a moving target depending on both the retailer’s own actions, and the actions of their competitors.* |

* Original START map factors with updated definitions

**^Ɨ^** A stock builds and reduces slowly over time relative to the factors in the map. If stock inputs were to decrease, the stock itself would retain its magnitude, depending on other outputs and inputs.
